# Supplementary material for: Rapid fluctuations in histamine associated with intake of nutritive and non-nutritive solutions
Source: bioRxiv. 2025 Mar 20:2024.11.07.622425. Preprint. [Version 3] doi: 10.1101/2024.11.07.622425 (PMC11908229; doi:10.1101/2024.11.07.622425)
Supplement: 1 [file NIHPP2024.11.07.622425V3-supplement-1.pdf]

bioRxiv preprint doi: https://doi.org/10.1101/2024.11.07.622425; this version posted March 20, 2025. The copyright holder for this preprint (which was not certified by peer review) is the author/funder, who has granted bioRxiv a license to display the preprint in perpetuity. It is made available under aCC-BY 4.0 International license.

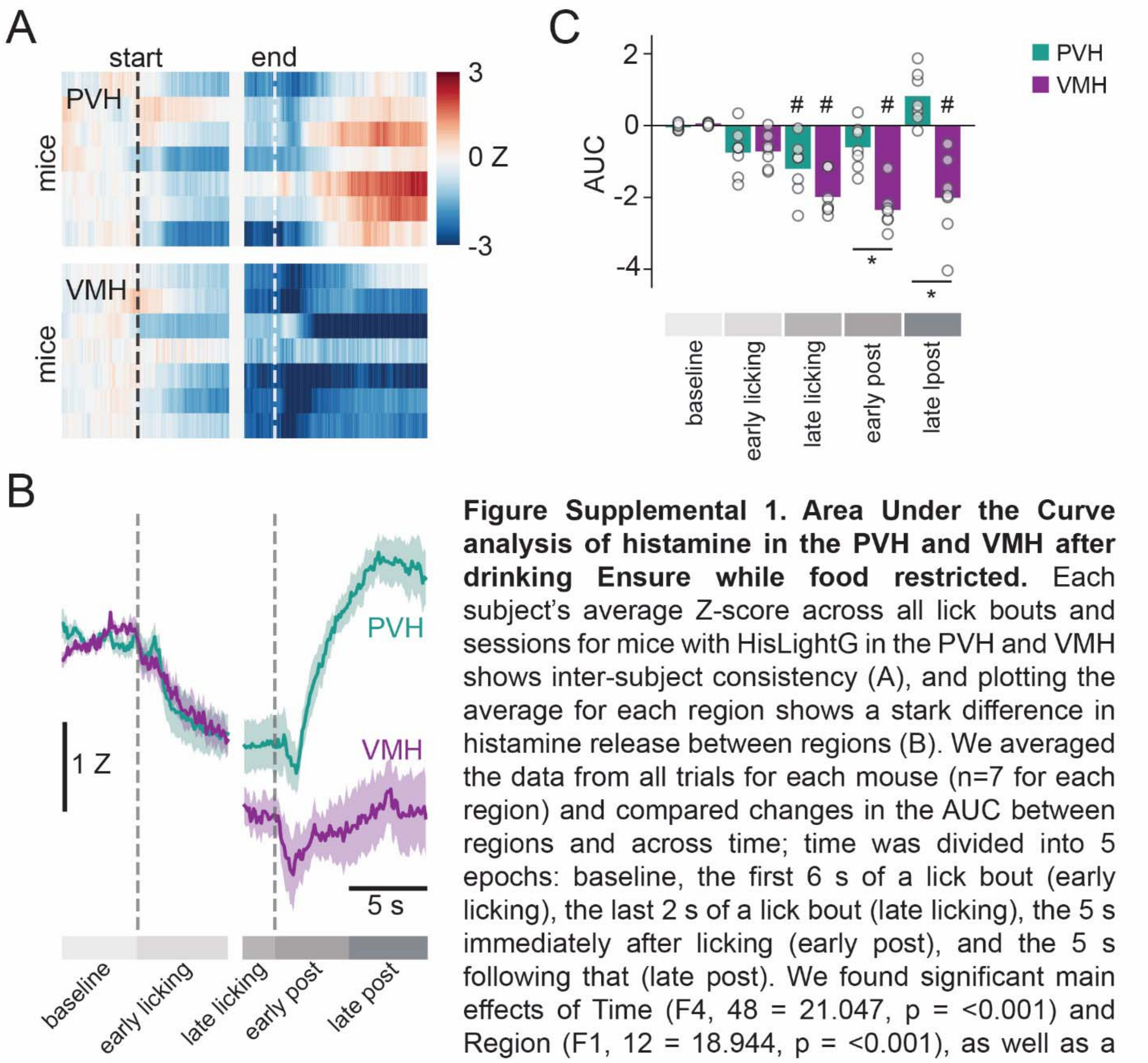

Figure Supplemental 1. Area Under the Curve analysis of histamine in the PVH and VMH after drinking Ensure while food restricted. Each subject's average Z-score across all lick bouts and sessions for mice with HisLightG in the PVH and VMH shows inter-subject consistency (A), and plotting the average for each region shows a stark difference in histamine release between regions (B). We averaged the data from all trials for each mouse (n=7 for each region) and compared changes in the AUC between regions and across time; time was divided into 5 epochs: baseline, the first 6 s of a lick bout (early licking), the last 2 s of a lick bout (late licking), the 5 s immediately after licking (early post), and the 5 s following that (late post). We found significant main effects of Time ($F4, 48 = 21.047$, $p = <0.001$) and Region ($F1, 12 = 18.944$, $p = <0.001$), as well as a significant interaction between the two ($F4, 48 = 18.518$, $p = <0.001$). Holm-corrected post hoc tests revealed that in the PVH, only the late licking epoch had a lower AUC than the baseline epoch, while in the VMH, the late licking, early post, and late post epochs all had lower AUCs than baseline. Furthermore, in both post-licking epochs, the AUC of PVH and VMH differed from one another (C). Bars are means and circles individual data points, # $p < 0.05$ from baseline for that region, * $p < 0.05$ between regions for that epoch.
